# Intravenous thrombolysis prevents neurological deterioration in patients with acute pontine infarction

**DOI:** 10.3389/fneur.2025.1462372

**Published:** 2025-01-22

**Authors:** Zhenxiao Chai, Weili Chen, Yichan Ye, Mengwan Song, Lingling Lin, Dongdong Lin, Xuerong Huang, Lifen Chi, Ruyue Huang

**Affiliations:** Department of Neurology, The Third Affiliated Hospital of Wenzhou Medical University, Ruian, Zhejiang, China

**Keywords:** infarction, pons, intracranial hemorrhagic transformation, intravenous thrombolysis, neurological deterioration

## Abstract

**Objectives:**

Neurological deterioration (ND) is common after acute isolated pontine infarction, and no evidence-based treatment is available to prevent this. We determined whether intravenous thrombolysis (IVT) with tissue plasminogen activator soon after pontine infarction prevents ND.

**Methods:**

We retrospectively enrolled consecutive patients admitted to our hospital within 4.5 h after the onset of isolated pontine infarction identified using diffusion-weighted imaging. Patients were divided into the IVT and non-IVT groups. ND was defined as any ≥2-point increase in the National Institutes of Health Stroke Scale (NIHSS) score between the maximal and initial neurological deficits during hospitalization. Patients' clinical characteristics, laboratory findings, and outcomes were analyzed to determine the efficacy and safety of IVT.

**Results:**

Of 211 study patients (median age, 67 years [interquartile range, 57–75 years]; 132 [62.6%] men), 74 received IVT; 137 patients did not receive IVT, but accepted other antithrombotic therapies, including antiplatelet or anticoagulant drugs. The NIHSS score on admission was higher in the IVT group than in the non-IVT group (7 vs. 4, *P* = 0.000), but that at discharge was similar in both groups (4 vs. 5, *P* = 0.975). ND occurred in 17 (23.0%) and 51 (37.2%) patients in the IVT and non-IVT groups, respectively (*P* = 0.044). Multiple logistic regression analysis identified IVT (odds ratio, 1.509; 95% confidence interval, 1.250–3.034) as an independent factor for preventing ND. The incidence of intracranial hemorrhagic transformation (*P* = 0.351) and major visceral organ hemorrhage (*P* = 0.122) was similar in both groups.

**Conclusions:**

IVT may prevent early ND after acute pontine infarction without increasing intracranial hemorrhagic transformation, possibly by decreasing the total thrombotic burden.

## Introduction

Isolated pontine infarction accounts for approximately 7% of all ischemic stroke (1) and approximately 15% of all posterior-circulation stroke (2). Neurological deterioration (ND), that is, the worsening of neurological symptoms within the first few days after onset, is relatively common in acute pontine infarctions. The incidence of ND ranges from 14% to 35% in different reports, depending on the diagnostic criteria used for ND and the interval between symptom onset and evaluation (2–6). ND is frequently associated with increased functional disability (4, 7, 8). After a stroke, ND can occur because of disruption of function in the infarcted area, due to mechanisms such as a propagating thrombus arising from an atheromatous lesion in the basilar artery, narrowing of arterial stenosis, development of brain edema, recurrent artery-to-artery embolism, and failure of collateral circulation (9). Moreover, the incidence of ND after pontine infarction remains high despite the early use of antiplatelet or anticoagulant drugs (4, 10, 11).

Intravenous recombinant tissue plasminogen activator (t-PA) administration is the standard reperfusion therapy for acute ischemic stroke within 4.5 h of onset; t-PA induces thrombus dissolution by activating plasminogen into plasmin (12). Thus, t-PA injection may be a potential treatment to prevent ND in patients with pontine infarction when administered in the very early stage after onset. Siegler et al. (13) reported that intravenous thrombolysis (IVT) attenuated ND after ischemic stroke. However, the relationship between thrombolytic therapy and ND in specific infarction areas has not been studied. Therefore, in this study, we aimed to determine the potential efficacy and safety of IVT with t-PA for preventing ND in patients with pontine infarction during the acute phase. The results presented in the present report may lead to the development of strategies to better manage patients with pontine infarction and improve clinical outcomes.

## Methods

### Patient selection

We retrospectively analyzed the demographic, clinical, laboratory, and radiographic data of consecutive patients who had isolated pontine infarction diagnosed using diffusion-weighted imaging (DWI) and were admitted to the stroke center in the Third Affiliated Hospital of Wenzhou Medical University between January 2012 and May 2023.

The inclusion criteria for this study were as follows: (1) admission to our hospital within 4.5 h of symptom onset and (2) acute ischemic lesions within the pons detected on DWI performed after admission. The exclusion criteria were as follows: (1) incomplete information, (2) duration of hospitalization < 3 days, (3) severe sequelae of previous stroke or brain injury affecting the judgment of ND, and (4) pontine infarction combined with other ischemic brain lesions. All patients with pontine infarction signed informed consent forms before undergoing thrombolysis. Our ethics committee of the Third Affiliated Hospital of Wenzhou Medical University approved this study (approval No. YJ2023053) and waived the need for informed consent due to its retrospective nature.

### Data collection and neuroimaging protocol

Demographic features and risk factors for infarction were investigated, including age, sex, smoking, drinking, prior infarction, atrial fibrillation, hypertension, and diabetes mellitus. We recorded the blood pressure taken at admission. The results of laboratory tests performed at the time of admission, including blood glucose, blood lipids (triglycerides, total cholesterol, and low-density lipoprotein), routine blood tests (hemoglobin, hematocrit, and platelets), and blood coagulation profile (international normalized ratio, and fibrinogen) were also collected from the patients' hospital records. All patients complied with medical advice to undergo magnetic resonance imaging (MRI; 1.5 T, Magnetom Avanto, Siemens, Germany) during hospitalization to identify the ischemic lesion in the pons. DWI was performed using 2 levels of diffusion sensitization (b values of 0 and 1,000 s/mm^2^, respectively, and 5-mm slice thickness with 1.5-mm gap). Post-treatment imaging was performed using computed tomography (CT) at 24 h after IVT initiation. Additional scans were performed at any time when ND occurred. The potential causes of isolated pontine infarction included 5 stroke subtypes (14): (1) vertebrobasilar large-artery disease (VLAD) was indicated by stenosis of at least 50% of the luminal diameter of the basilar artery, as determined via magnetic resonance angiography or CT angiography; (2) basilar artery branch disease (BABD) was indicated by an infarct that reached the pontine surface in the absence of large-artery disease and other potential sources; (3) small-artery disease (SAD) was indicated by a small (< 15 mm) infarct that spared the surface of the pons in the absence of other etiologies, as assessed by MRI; (4) cardioembolism (CE) was indicated by potential cardiac sources of embolism, and included mainly atrial fibrillation; and (5) other and undetermined causes.

### Clinical information and assessment

In our study, both progressive motor deficits as well as worsening of other symptoms such as dysarthria, ataxia, and facial palsy were considered as clinical manifestations of ND. To quantify the clinical progression of the patients' neurological symptoms, we measured their National Institutes of Health Stroke Scale (NIHSS) score at the time of admission, at the time of maximal neurological deficit (if this occurred during hospitalization), and at the time of discharge. Among patients who underwent IVT, we also recorded the NIHSS score at 2 h after IVT. ND was defined as any ≥2-point increase in the total NIHSS score between the maximal and initial neurological deficits (11, 15, 16). Any bleeding events that occurred during hospitalization were recorded. The pre-specified bleeding events were intracranial hemorrhagic transformation, mucocutaneous hemorrhage, and major visceral organ hemorrhage. Intracranial hemorrhagic transformation was defined according to the European Cooperative Acute Stroke Study III criteria (17).

### Statistical analysis

The Student *t*-test or Mann-Whitney rank-sum test was used to evaluate continuous variables, which were presented as mean ± standard deviation or median (interquartile range, IQR). The chi-squared or Fisher exact test was used to assess non-continuous variables, which were presented as frequencies and percentages. Multivariate logistic regression analysis was performed to identify independent factors for preventing ND. Variables from the univariate analyses with *P* values < 0.1 were considered to represent explanatory variables and entered into the multivariate analysis. All statistical analyses were performed using SPSS *v*26.0 statistical software (IBM, Chicago, IL). A *P* value of < 0.05 was considered to indicate a significant difference.

## Results

### General patient information

Among 25,100 patients who admitted for acute ischemic stroke to our hospital during the study period, 1984 (7.9%) patients were diagnosed with acute isolated pontine infarction on DWI. Of them, 240 patients with pontine infarction were admitted to the hospital within 4.5 h after symptom onset. Of these 240 patients, 29 patients were sequentially excluded due to the following reasons: (1) 25 patients had incomplete information, (2) two patients were hospitalized for < 3 days, and (3) two patients had severe sequelae of previous stroke or brain injury affecting the judgment of ND. Thus, a total of 211 eligible patients, consisting of 132 (62.6%) men and 79 (37.4%) women, with a median age of 67 years (IQR: 57–75 years) were included in this retrospective analysis. Of them, 74 patients received IVT, while 137 patients who were admitted to the hospital within 4.5 h after stroke onset chose not to receive IVT mainly because of concerns about bleeding complications, minor stroke, rapid improvement, or economic difficulties.

No significant differences were found between the IVT and non-IVT groups in terms of demographic characteristics (sex and age), risk factors (smoking tobacco, drinking alcohol, hypertension, diabetes mellitus, atrial fibrillation, and prior infarction), blood pressure at admission, and laboratory findings (blood glucose level, blood lipid level, and routine blood test results; *P* > 0.05, Table 1). VLAD was significantly more frequent in the IVT group (39.2%) than in the non-IVT group (23.4%, *P* = 0.018, Table 1).

**Table 1:** Baseline characteristics of patients with isolated pontine infarction.

**Variable**	**IVT group**	**Non-IVT group**	***P* value**
	**(*n =* 74)**	**(*n =* 137)**	
Age, y	64.5 (55–71)	68 (59–76)	0.065
Male sex, *n* (%)	50 (67.6)	82 (59.9)	0.918
Smoking tobacco, *n* (%)	25 (33.8)	45 (32.8)	0.89
Drinking alcohol, *n* (%)	19 (25.7)	38 (27.7)	0.724
Prior infarction, *n* (%)	11 (14.9)	19 (13.9)	0.843
Atrial fibrillation, *n* (%)	2 (2.7)	7 (5.1)	0.409
Hypertension, *n* (%)	68 (91.9)	127 (92.7)	0.832
Diabetes, *n* (%)	33 (44.6)	68 (49.6)	0.484
Systolic blood pressure, mm Hg	167.5 (156–179)	165 (151–181)	0.607
Diastolic blood pressure, mm Hg	85 (79–96)	85 (74–95)	0.252
Glucose, mmol/L	7.31 (5.71–10.64)	7.75 (5.78–10.73)	0.561
Triglycerides, mmol/L	1.27 (0.99–1.67)	1.42 (0.98–2.05)	0.103
Total cholesterol, mmol/L	4.69 (4.12–5.53)	4.83 (3.84–5.56)	0.887
Low-density lipoprotein, mmol/L	3.01 (2.37–3.68)	2.98 (2.44–3.79)	0.551
Hemoglobin, g/L	136 (121–145)	131 (122–140)	0.284
Hematocrit, %	0.40 (0.36–0.43)	0.38 (0.36–0.41)	0.117
Platelet (10^9^/L)	201 (169–247)	215 (178–259)	0.125
International normalized ratio	0.99 (0.92–1.04)	0.98 (0.92–1.03)	0.409
Fibrinogen, g/L	2.71 (2.39–3.67)	3.14 (2.51–3.83)	0.105
Hospital stay, days	14 (10.5–17)	14 (10–17)	0.554
**Stroke subtypes**, ***n*** **(%)**			
VLAD	29 (39.2)	32 (23.4)	0.018^*^
BABD	31 (41.9)	69 (50.4)	0.39
SAD	11 (14.9)	33 (24.1)	0.155
CE	1 (1.4)	3 (2.2)	0.67
Other and undetermined causes	2 (2.7)	3 (2.2)	0.815
**NIHSS score**			
On admission	7 (4–9)	4 (3–6)	0.000^*^
At discharge	4 (2–6.5)	5 (3–6)	0.975

IVT, intravenous thrombolysis; NIHSS, National Institutes of Health Stroke Scale; VLAD, vertebrobasilar large-artery disease; BABD, basilar artery branch disease; SAD, small-artery disease; CE, cardioembolism. 1 mm Hg = 0.133 kPa; ^*^denotes *P* value < 0.05.

### Antithrombotic treatments

All 74 patients in the IVT group received 0.9 mg/kg alteplase after admission. Apart from one patient with hemorrhagic transformation and two patients with visceral organ hemorrhage after IVT, 71 patients also received antiplatelet or anticoagulant therapy after routine re-examination of head CT scans at 24 h after IVT. The specific medications were as follows: 35 (49.3%) patients received oral dual antiplatelet therapy (loading dose of aspirin plus clopidogrel); 35 (49.3%) patients received single antiplatelet therapy (aspirin, clopidogrel, or tirofiban); and one (1.4%) patient was given anticoagulant therapy (low-molecular-weight heparin). In the non-IVT group, most patients received antithrombotic drugs after admission. Specifically, 56 (40.9%) patients received oral dual antiplatelet therapy (loading dose of aspirin plus clopidogrel); 75 (54.7%) patients received single antiplatelet therapy (aspirin, clopidogrel, or tirofiban); three (2.2%) patients were given anticoagulant therapy (low-molecular-weight heparin); and three (2.2%) patients did not receive any antithrombotic drugs.

### Neurological deterioration

**Figure 1:**
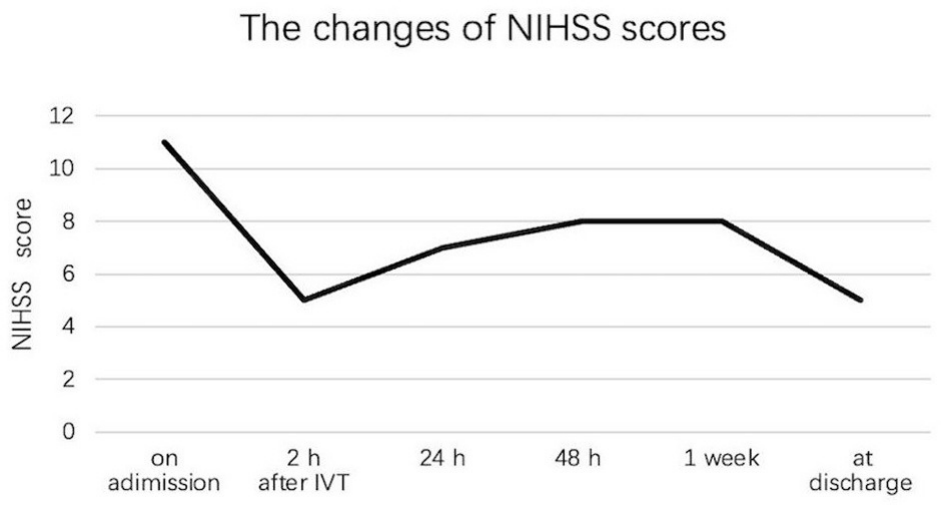
Neurological deficit fluctuation among one patient with isolated pontine infarction after IVT during hospitalization.

Of the total of 211 patients, 68 (32.2%) patients developed ND during hospitalization, including 17 (23.0%) patients in the IVT group and 51 (37.2%) patients in the non-IVT group. The incidence of ND was significantly higher in the non-IVT group than in the IVT group (*P* = 0.044, Table 2). In the IVT group, 9 patients had transient neurological improvement at 2 h after IVT, but worsened subsequently. Because the difference in the NIHSS score at the time of the maximal and initial neurological deficits was < 2 points, these 9 patients were not diagnosed with ND. One representative case with neurological deficit fluctuation after t-PA infusion is shown in Figure 1.

**Table 2:** Incidence of ND in patients with isolated pontine infarction.

**Variable**	**IVT group (*n =* 74)**	**Non-IVT group (*n =* 137)**	***P* value**
Rate of ND, *n* (%)	17 (23.0)	51 (37.2)	0.044
	**Patients with ND (*****n*** = **17)**	**Patients with ND (*****n*** = **51)**	
**Time distribution of ND**, ***n*** **(%)**			
≤ 2 days after stroke onset	16 (94.1)	44 (86.3)	0.385
>2 days after stroke onset	1 (5.9)	7 (13.7)	
**Degree of ND (**Δ **NIHSS score)**			
	3 (3–5.5)	3 (2–5)	0.476

IVT, intravenous thrombolysis; ND, neurological deterioration; NIHSS, National Institutes of Health Stroke Scale.

**Figure 2:**
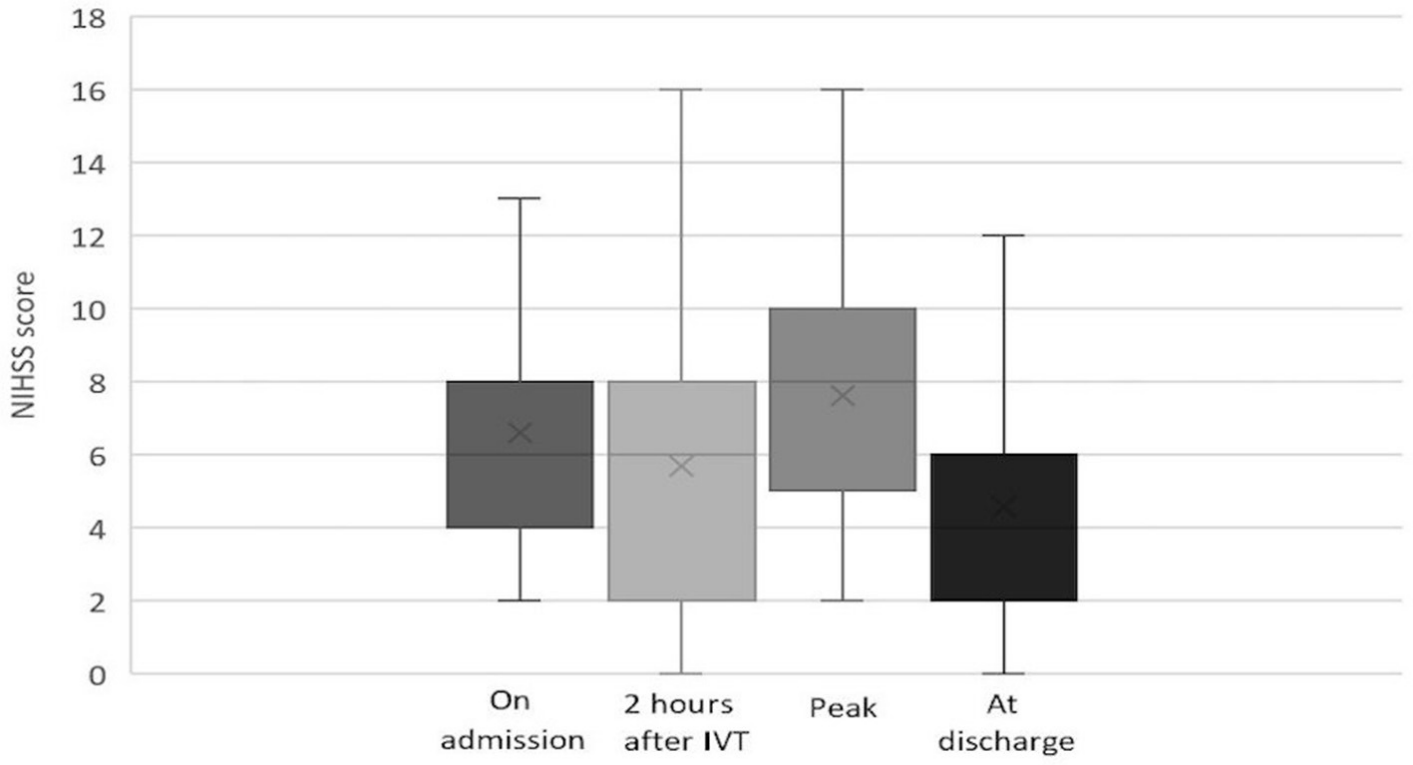
Evolution of NIHSS score in pontine infarction patients with IVT.

**Figure 3:**
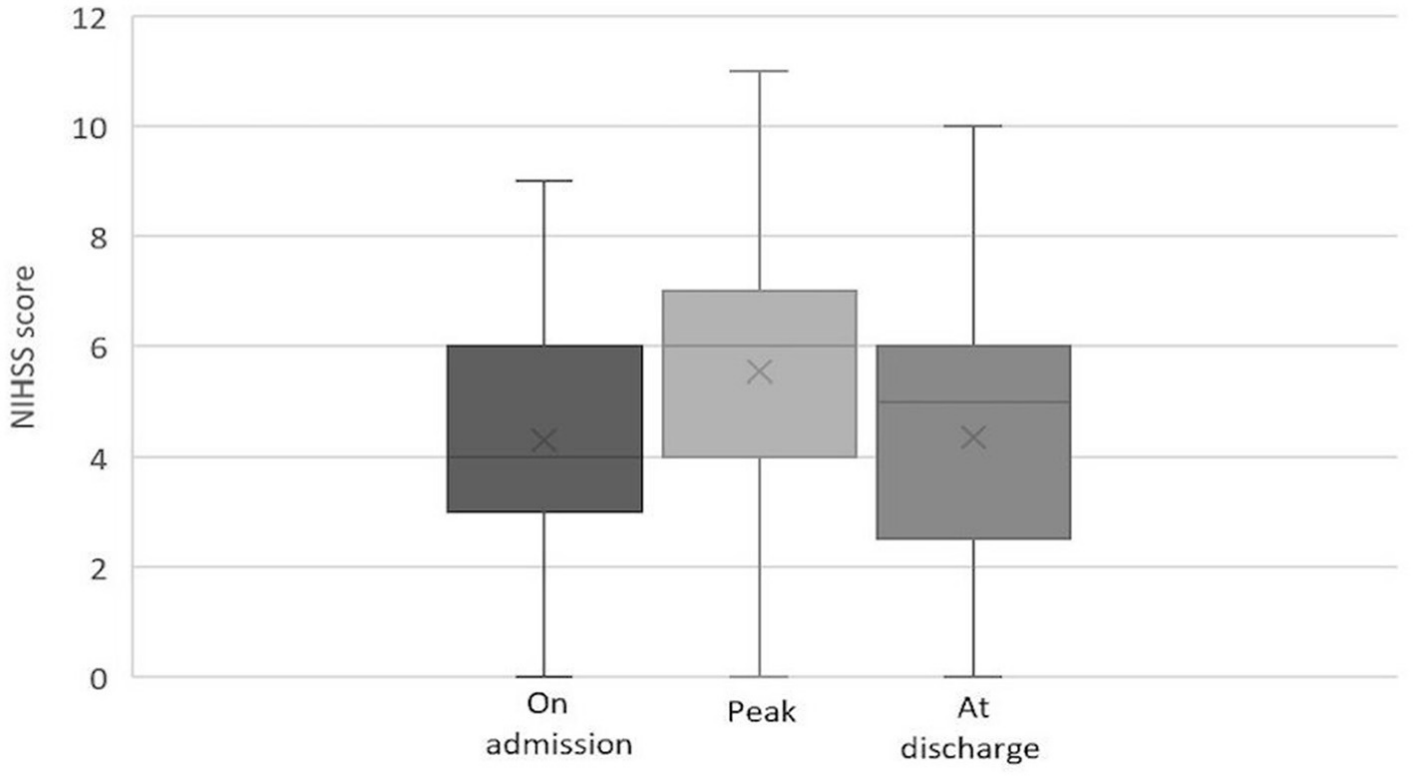
Evolution of NIHSS score in pontine infarction patients without IVT.

Among patients with IVT, the median NIHSS score was 7 (IQR: 4–9) on admission, 6 (IQR: 2–8) at 2 h after IVT, 8 (IQR: 5–10) at maximum, and 4 (IQR: 2–6.5) at discharge. The evolution of NIHSS scores in the IVT group is depicted in Figure 2. Among patients without IVT, the median NIHSS score was 4 (IQR: 3–6) on admission, 6 (IQR: 4–7.5) at maximum, and 5 (IQR: 3–6) at discharge. The evolution of NIHSS scores in the non-IVT group is depicted in Figure 3. The median NIHSS score on admission was higher in patients with IVT than in those without IVT (7 vs. 4, *P* = 0.000), while there was no difference in the NIHSS score at discharge between the two groups (4 vs. 5, *P* = 0.975, Table 1).

Of the 17 patients with ND in the IVT group, 16 (94.1%) developed deterioration within 48 h, while of the 51 patients with ND in the non-IVT group, 44 (66.3%) developed ND within 48 h. No significant difference was observed in the time distribution of ND between the two groups (*P* = 0.385, Table 2).

The Δ NIHSS (maximal NIHSS minus initial NIHSS) was used to ascertain the degree of ND. The Δ NIHSS was 3 (IQR: 3–3.5) in the IVT group and 3 (IQR: 2–5) in the non-IVT group. No significant difference in the degree of ND was observed between the two groups (*P* = 0.476, Table 2).

Multiple logistic regression analysis was conducted to further evaluate independent factors for preventing ND. The results showed that IVT (odds ratio, 1.509; 95% confidence interval, 1.250–3.034) was an independent factor for preventing ND (Table 3).

**Table 3:** Multivariate logistic regression analysis of factors potentially related to the prevention of ND in patients with isolated pontine infarction.

	**Multivariate OR (95% CI)**	***P* value**
Age	0.998 (0.990–1.006)	0.597
VLAD	1.546 (0.800–2.986)	0.195
NIHSS score on admission	0.959 (0.861–1.068)	0.444
IVT	1.509 (1.250–3.034)	0.042

ND, neurological deterioration; OR, odds ratio; CI, confidence interval; VLAD, vertebrobasilar large-artery disease; NIHSS, National Institutes of Health Stroke Scale; IVT, intravenous thrombolysis.

### Bleeding complications

Bleeding events occurred in both groups, including intracranial hemorrhagic transformation, mucocutaneous hemorrhage, and visceral organ hemorrhage (Table 4). The proportion of intracranial hemorrhagic transformation was one of 74 (1.4%) in the IVT group and 0 of 137 (0%) in the non-IVT group (*P* = 0.351, Table 4). The only patient with intracranial hemorrhagic transformation after IVT had no clinical ND.

**Table 4:** Hemorrhagic complications among patients with pontine infarction.

**Variable**	**IVT group (*n =* 74)**	**Non-IVT group** ** (*n =* 137)**	***P* value**
Hemorrhagic transformation, *n* (%)	1 (1.4)	0 (0)	0.351
Mucocutaneous hemorrhage, *n* (%)	21 (29.2)	2 (1.5)	0.000
Visceral organ hemorrhage, *n* (%)	2 (2.7)	0 (0)	0.122

IVT, intravenous thrombolysis.

The incidence of mucocutaneous hemorrhage was significantly higher in patients with IVT than in those without IVT (29.2% vs. 1.5%, *P* = 0.000, Table 4). In the IVT group, 17 of the 21 (81.0%) patients developed gingival bleeding, and four of the 21 (19.0%) patients developed nasopharyngeal mucosal bleeding. All instances of mucocutaneous hemorrhage stopped spontaneously or after the application of pressure with a cotton swab. In the non-IVT group, only two patients developed gingival bleeding.

Visceral organ hemorrhage occurred in a total of two patients in the IVT group. One of these patients experienced upper gastrointestinal bleeding, which stopped after treatment with proton pump inhibitors. The other patient developed severe renal hemorrhage with a decrease in hemoglobin level to 44 g/L; this patient was transfused with a total of 3.5 units of packed red blood cells. No instances of visceral organ hemorrhage occurred in the non-IVT group. The incidence of visceral organ hemorrhage did not significantly differ between the 2 groups (*P* = 0.122, Table 4).

## Discussion

The present study revealed that IVT with t-PA in the early stage after isolated pontine infarction was associated with a decreased incidence of ND, as compared with patients who received other types of antithrombotic therapies. Although the median NIHSS score on admission was higher in the IVT group than in the non-IVT group, the NIHSS score at discharge did not differ between the two groups. In the IVT group, one patient developed intracranial hemorrhagic transformation, and two patients developed visceral organ hemorrhage; however, the incidence of these two complications did not significantly differ between the IVT and non-IVT groups.

Many definitions of ND have been used in previous studies, depending on the stroke scale used to assess deterioration, the degree of worsening, and the time frame of the deterioration (18, 19). Progression of neurological deficits is relatively common in patients with acute isolated pontine infarction. In the present study, 68 (32.2%) patients with acute pontine infarction developed ND, which is higher than the rate reported by Li et al. (28.0%) (20). This difference might be attributable to the earlier admission (within 4.5 h after stroke onset) in our study, which was helpful to investigate the whole clinical course, especially early symptom deterioration. Vynckier et al. (15) have reported that permanent or non-permanent deterioration of only one point on the NIHSS should not be considered ND because this is within the expected interrater variability of the NIHSS for lacunar strokes. Some researchers have found that Δ NIHSS ≥ 2 points could be used to define ND in specific studies on minor strokes, where even small deteriorations have clinical significance (19, 21). The clinical presentations of pontine infarction are usually mild, and ischemic lesions of the pons are lacunar (4, 10). The median NIHSS score on admission of all our patients was 5. Therefore, in our study, ND was defined as any increase of ≥2 points in the total NIHSS score between the maximal and initial neurological deficits after admission, which is more representative of the progression of pontine infarction.

Apart from straightforward causes, such as intracerebral hemorrhage and malignant edema, the mechanism of ND remains mostly unclear. *In situ* extension of the original thrombus in the same territory is a promising hypothesis to explain secondary hemodynamic compromise, via occlusion of previously unaffected perforators, branches, or collaterals (21). Early in 1996, Bassetti et al. (2) proposed that in patients with isolated infarcts of the pons, ND might be caused by diminished perfusion arising from atheromatous lesions at the orifices of large-caliber penetrating arteries or propagating thrombosis from atheromatous lesions. Recently, a study found that unexplained ND occurring after thrombolysis was independently associated with extension of the susceptibility vessel sign on MRI, suggesting that unexplained ND may be associated with thrombus extension (22). Thus, the formation and alteration of a thrombus might be a major pathophysiological mechanism driving the occurrence and evolution of ischemic infarction. However, intensive antithrombotic treatments, including antiplatelet or anticoagulation therapy, failed to reverse ND (10, 11, 20). Consistent with this, the incidence rate of ND in our study was found to be high (37.2%) in patients who did not receive IVT with t-PA, despite aggressive antithrombotic therapy. Rossi et al. (23) demonstrated that the administration of thrombolytic agents significantly reduces thrombus size, and reduces all the main histological components of the thrombus (platelets, red blood cells, and fibrin). We speculated whether thrombolytic therapy with t-PA in the early stage after stroke onset could be the best approach to prevent ND in patients with acute pontine infarction. As a major result of our study, we found that the incidence of ND was significantly lower in the IVT group than in the non-IVT group, and IVT was an independent factor for preventing ND in patients with isolated pontine infarction. In the IVT group, 17 (23.0%) patients experienced ND, whereas 48 (64.9%) remained relatively neurologically stable, and 9 (12.1%) had transient fluctuations of clinical symptoms. The nine patients with transient fluctuations displayed rapid improvement at 2 h after IVT, then deteriorated mostly within 24 h. Because the difference in their NIHSS scores at the maximal and initial neurological deficits was < 2 points, these nine patients were not diagnosed with ND. We speculated that in the patients with transient fluctuations, t-PA might have dissolved the blood clot rapidly after intravenous injection, but then, the thrombus extended to some extent, though not up to the initial level. Therefore, we inferred that IVT with t-PA could prevent ND by decreasing the total thrombotic burden.

In the present study, we found that the initial NIHSS score was significantly higher and VLAD was more frequent in acute pontine infarction patients with IVT than in patients without IVT, which might be interpreted as large-artery atherosclerosis being associated with the highest residual disability (24). This indicated that physicians were more cautious about administering IVT to pontine infarction patients who had mild neurological deficits in the early stage, owing to concerns about major bleeding complications. Hemorrhagic transformation following pontine infarction can be a potentially devastating condition (25). All the patients with ND were re-examined by head imaging. Only one of the 74 (1.4%) patients in the IVT group developed intracranial hemorrhagic transformation, while none of the 137 patients in the non-IVT group developed intracranial hemorrhagic transformation. This observation is in line with the results of an international prospective observational registry study of basilar artery occlusion, which reported that hemorrhagic transformation occurred in only 0.5% (1/183) of patients with brainstem infarction who were treated with either anticoagulants or antiplatelet agents (26). The risk of hemorrhagic transformation increases with the use of t-PA in acute ischemic stroke (27), but we found no significant difference in the proportion of hemorrhagic transformation between pontine infarction patients with and without IVT. The following hypotheses might explain why hemorrhagic transformation seldom occurs in brainstem infarction. First, pontine infarcts tend to be small, and large infarcts are a risk factor for hemorrhagic transformation. Second, the brainstem is well supplied by small end-arteries with collateral flow, which may result in the slower evolution of irreversible ischemia in posterior-circulation strokes with proximal artery occlusion (28). It should be noted that upper gastrointestinal bleeding and serious renal hemorrhage occurred in 2 patients in our study, and thus, clinicians should be very cautious about major visceral organ hemorrhage when administering IVT to patients with pontine infarction.

Our study offers valuable insights on several fronts. First, no study has as yet assessed the incidence of ND in patients undergoing recanalization therapy for pontine infarction. This is the first study to demonstrate the efficacy of IVT with t-PA for preventing ND in patients with acute pontine infarction. Second, we studied the details of ND in the early stage of stroke onset, which might provide specific practice guidance for clinicians to make decisions about administering thrombolysis to patients with acute pontine infarction, especially those with mild deficits in the early stage after onset.

There are several limitations to this study. First, this was a retrospective study with a modest sample size. We only analyzed the efficacy and safety of IVT to prevent ND in patients with pontine infarction. A larger sample size should be followed in future studies to prove if IVT can result in better functional outcomes at 3 months. Second, repeat MRI was not performed after ND in the majority of patients, and the exact cause of progression could not be confirmed using imaging studies. Third, the statistical analysis in the present study is relatively simple, but it is still useful because of the urgent need for this research.

## Conclusion

In conclusion, this is the first study to demonstrate the efficacy of IVT with t-PA for preventing ND in patients with acute pontine infarction. We found that IVT could prevent ND in patients with acute pontine infarction by decreasing the total thrombotic burden. Despite having higher NIHSS scores at the baseline, the IVT group had similar NIHSS scores as the non-IVT group at discharge, suggesting that IVT prevented function decline in patients with acute pontine infarction. Additionally, multivariate logistic regression analysis identified IVT as an independent factor for preventing ND. Furthermore, IVT did not increase the incidence of major bleeding events, which indicated that the administration of IVT to these patients appeared to be safe. Therefore, clinicians should recommend IVT for patients with suspected pontine infarction, even if their neurological deficits are mild within 4.5 h after onset.

## Data Availability

The raw data supporting the conclusions of this article will be made available by the authors, without undue reservation.
